# Cushing Syndrome due to Inappropriate Corticosteroid Topical Treatment of Undiagnosed Scabies

**DOI:** 10.3390/tropicalmed3030082

**Published:** 2018-08-03

**Authors:** Guadalupe Estrada-Chávez, Roberto Estrada, Daniel Engelman, Jesus Molina, Guadalupe Chávez-López

**Affiliations:** 1 Department of Dermatology and Dermato-Oncology, Instituto Estatal de Cancerología “Dr. Arturo Beltrán Ortega”, Health Secretary Guerrero, Faculty of Medicine, Universidad Autónoma de Guerrero Mexico, Community Dermatology Mexico C.A., 39850 Acapulco, Guerrero, Mexico; estradaguadalupe@hotmail.com; 2 Community Dermatology Mexico C.A.; Health Secretary Guerrero, 39355 Acapulco, Guerrero, Mexico; restrada_13@hotmail.com; 3 Centre for International Child Health, University of Melbourne, Melbourne, Australia/Group A Streptococcal Research, Murdoch Children’s Research Institute, 3010 Melbourne, Australia; Daniel.Engelman@rch.org.au; 4 Department of Pediatrics, Acapulco General Hospital, Health Secretary Guerrero, 39901 Acapulco, Guerrero, Mexico; molinabravo@prodigy.net.mx; 5 Department of Dermatology and Mycology Acapulco General Hospital, Health Secretary Guerrero, Community Dermatology Mexico C.A., 39355 Acapulco, Guerrero, Mexico

**Keywords:** scabies, Cushing syndrome, iatrogenic, topical corticosteroids

## Abstract

The uncontrolled sale of topical corticosteroids has become an important risk factor for the development of iatrogenic Cushing syndrome in children, especially in countries where medications are sold over the counter. This is exacerbated by the lack of information for both the patients and pharmacists. This report documents a series of eight cases of iatrogenic Cushing syndrome secondary to an inappropriate use of topical steroids, due to a misdiagnosis of scabies.

## 1. Introduction

Scabies is a highly contagious skin disease caused by the mite *Sarcoptes scabiei* var. *hominis.* Transmission mainly occurs through direct skin contact, often from family members. Scabies is estimated to affect 200 million people worldwide [1]. Reported prevalence rates vary from 0.3–46% [2], with the highest prevalence rate observed in children in tropical countries. Predisposed factors include heat, humidity, overcrowding and increased contact with exposed skin areas [3]. Common (also known as typical or classical) scabies is highly symptomatic, with an intense itch and a rash consisting of discrete to moderate disseminated papules. These are secondary to the antigen released by the parasite and subsequent immune and inflammatory reactions. Other family members frequently have similar symptoms. Crusted scabies is a rarer form of the disease, usually affecting immunosuppressed patients, and presents with hyperkeratotic skin lesions containing thousands of mites.

In Mexico, a common superstition suggests that infants can develop illness if they are not held by a person who gazes at them, as it puts the infant at risk of acquiring ‘the evil eye’. Therefore, parents commonly encourage others, even complete strangers, to touch and hold their child [4]. There is also a general lack of awareness that prolonged contact with an infected person can be the cause of scabies or other infectious diseases. Instead, dogs, cats or poor quality of water are often blamed as the cause of the disease. Once a child becomes unwell, parents commonly use household remedies or medications obtained at local pharmacies, where employees may not have any medical or pharmacological training. As many inflammatory dermatological conditions will respond to topical corticosteroids, these are frequently purchased by patients without consultation with a clinician. Topical corticosteroids are known to exacerbate some conditions, particularly infections such as tinea and scabies [5,6].

Topical steroids are graded in terms of potency as either low, medium, high or very high. Whilst low potency steroids are often well tolerated, the absorption of higher potency steroids through the skin can cause Cushing syndrome due to suppression of the hypothalamic-pituitary-adrenal axis, especially if used over large areas of skin, or for prolonged periods [7,8]. In Mexico, topical steroids at all potencies are available without medical prescription over the counter. One of those most commonly sold products is a combination cream containing betamethasone (0.5%), clotrimazole, and gentamicin. The cost is approximately 1.5 USD per 25 g tube [9]. In recent times, high potency combination steroid creams have been widely promoted in Mexico on television, bus advertising, social media and on-site at pharmacies. Even though steroids are used widely in dermatology, their misuse for the treatment of scabies has become increasingly common and problematic in the last 2 to 3 years, causing severe medical problems that deserve wider recognition and prevention.

Permethrin is the most effective topical treatment for scabies, but, it is expensive, which limits its use in Mexico. Other topical treatments for scabies include benzyl benzoate (the most widely available in Mexico), sulfur cream or ointment, crotamiton and lindane [10]. Topical treatments frequently cause adverse effects, including contact dermatitis, erythema, and a worsening itch, which can lead to the early suspension of treatment, intermittent usage and a high frequency of relapse. Ivermectin is an effective oral treatment but is unavailable in many settings. Furthermore, although treatment of all close contacts is recommended, family members and other contacts may be asymptomatic or otherwise reluctant to be treated, further encouraging reinfestation [11].

## 2. Case Series 

This report documents a series of cases involving eight infants, aged from 3 months to 1.5 years (Table A1). All patients clinical signs of Cushing syndrome, including increased adiposity, prominent cheeks, mild to moderate hirsutism on the forehead and side brows, discrete striae and telangiectasias. One patient had such significant weight gain that she lost the ability to support her own body weight. All patients also had disseminated lesions of scabies, with papules, scaling and nodules predominantly in skin folds and the soles of their feet. Two infants had the severe form of crusted scabies. 

All cases had a history of continuous or discontinuous use of topical corticosteroids (betamethasone (0.5%) combined with clotrimazole and gentamicin), applied to affected lesions or used on the entire body’s surface. 

All scabies patients were diagnosed at the Dermatology department of the Acapulco General Hospital in Guerrero, Mexico. Diagnosis was made clinically in all patients based on the typical rash, itch and affected cohabitants. The patients with crusted scabies had extensive scaling lesions on the back, palms and soles with moderate papules and discrete nodules on the rest of the body’s surface, particularly in the skin folds. Symptoms of scabies had been present for 1 to 7 months prior to diagnosis. In all cases, the mothers also had clinical lesions of scabies, and in four of eight cases, the mothers had a history that was suggestive of scabies during pregnancy. The number of family members in each household that had daily contact with the patients ranged from 3 to 12. In all cases, the use of the steroid cream was reported to have resulted in initial improvement of symptoms. However, the signs and symptoms would return and worsen whenever the usage of corticosteroid was suspended. Affected family members did not seek medical advice until symptoms worsened. None of the parents were aware that the weight gain and other physical signs of Cushing syndrome were related to the use of the steroid cream. Two patients experienced recurrent upper pulmonary infections during the use of topical steroids that required referral to a physician. 

All patients were treated with sulfur cream (4%). Family contacts were treated with oral ivermectin. Twenty-four-hour urine cortisol was requested for patients, but this was often not completed in the majority of cases due to lack of financial resources. One case had the investigation performed one month after cessation of betamethasone, which was reported to be within normal limits. 

Follow-up information for patients was limited by the fact that families resided in distant places. All patients were referred to the pediatric service, while two attended for a consultation, with no further complications found. The rest of the patients did not attend a consultation, possibly due to the resolution of scabies symptoms after effective treatment and cessation of steroids. A telephone contact follow-up was attempted but unsuccessful. A check of the patients’ hospital files did not reveal any further visits for outpatient consultation or to the emergency department.

## 3. Discussion

**Figure A3:**
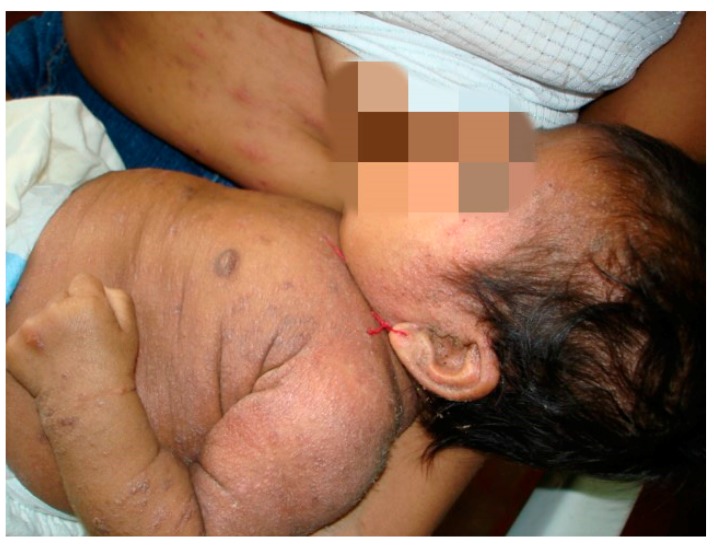
Crusted scabies with intense scaling and nodular lesions in child and mother with scabies.

Scabies has recently been added to the list of Neglected Tropical Diseases designated as priorities for control by the World Health Organization [12]. Organizations like the International Alliance for the Control of Scabies have focused on building the evidence base and advocacy strategy for the global control of scabies. This is because of the disease’s frequency, global burden, complications and effects on already disadvantaged populations [13]. Whilst common scabies, without secondary bacterial infection, is not life threatening and can be treated simply in many patients, this case series demonstrates that the uncontrolled and chronic use of high potency steroids, including clobetasol and betamethasone, can lead to potentially harmful complications such as Cushing syndrome and other endocrine disorders as well as the development of crusted scabies [7,14]. Patients with crusted scabies have intense scaling on the palms and soles and numerous nodular lesions on axillary and inguinal folds as well as on the scrotum for boys and diaper rim on the waist [15] (Figure A3). Topical or systemic glucocorticoids should be used with caution and never as a treatment for scabies in children.

There are many factors that contribute to the development of Cushing syndrome in this case series and the many other cases we have observed in our practice. Scabies is often not accurately diagnosed, particularly as a medical opinion is not sought after by families, which leads to erroneous treatment. Scabies has recently re-emerged as a major health problem in parts of Mexico, and families are searching for effective treatments for this highly symptomatic disease. At the same time, over-the-counter creams have been promoted to pharmacies and general practitioners as low-cost treatments for a broad range of bacterial, mycotic and inflammatory skin conditions. Previously, steroid-misuse complications have been widely reported, but most reports relate to either eczema in developed countries or to skin lightening creams or fungal infections in underdeveloped countries [5,16]. Patients with scabies may consider these treatments to be cost effective due to the rapid (initial) relief of itch and desire to avoid the cost of medical consultation [17]. 

Parents may not recognize the rapid weight gain as one of the signs of the infant’s developing health problem [4], due to the common belief that ‘a fat baby is a healthy one’, which leads to reassuring and positive comments on the infant’s appearance from friends and family members [18,19].

The incidence of Cushing syndrome secondary from the use of topical steroids is unknown and quite likely underreported [8,20,21,22]. In addition to the inappropriate treatment of scabies and other skin disorders (including diaper rash, psoriasis, tineas or inflammatory diseases) causing iatrogenic Cushing syndrome, there are reports of Cushing syndrome being caused by inappropriate steroid use for ophthalmologic conditions including cataracts and glaucoma [23]. These risks reinforce the importance of reporting the steroid-related consequences and secondary effects. Further reporting and investigation of these issues may increase awareness amongst health workers and organizations which will lead to advocacy for the regulation and restriction of cortisone-containing products in Mexico and other countries experiencing this issue [24,25,26].

Sulfur and benzyl benzoate are the most available topical scabies treatments in Mexico. Even though they are effective if applied to the whole body for the recommended duration, they frequently cause irritation, erythema, scaling, intense pruritus or even contact dermatitis, all of which may lead to the interruption of treatment or discontinuous therapy, which ultimately reduces the effectiveness of the treatment. Alternative treatments for scabies, such as tea tree oil, are under investigation but their effectiveness is unproven [27]. Economical and effective treatments are still needed in resource-limited countries, where family income is usually reserved for food, housing and clothing which leaves non-life-threating diseases as lower priorities [28].

The education of health workers is of great importance, as they are the point of first contact. Health workers need to know how to recognize, diagnose and appropriately treat common scabies, and to avoid the use of corticosteroids, especially in children. Complicated cases, or those that do not improve with first-line treatments, should be referred for further assessment. In countries like Mexico, in which there are fewer regulations on the sale of medications, it is essential to inform the general population of the potential risks when medications are not used as intended. 

We have introduced teledermatology as part of the Community Dermatology program in Mexico [29] for the education of health workers and doctors in isolated communities. This approach promotes improvements in clinical diagnosis and treatment and can be used to reduce the number of complicated cases. Greater awareness and action are needed for the widespread health problems related to the inappropriate use of steroids [30].

## 4. Ethical Considerations

The study protocol was approved by the Ethical Review Committee of the Community Dermatology A.C. and was supported by the Health Secretary of Guerrero State prior to the study. Informed consent was obtained from the parents or legal guardians of the minors after a detailed explanation of the study’s protocol. In accordance with the ethical review committee requirements, patient information was made confidential.

## Figures and Tables

**Figure A1:**
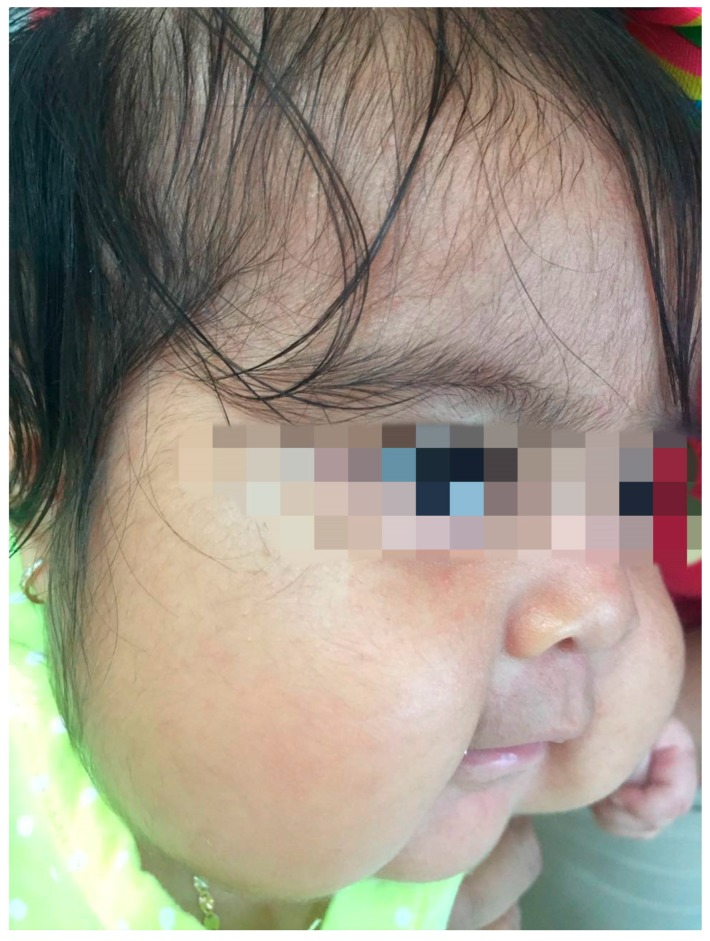
Infant with increased adiposity and forehead hirsutism.

**Figure A2:**
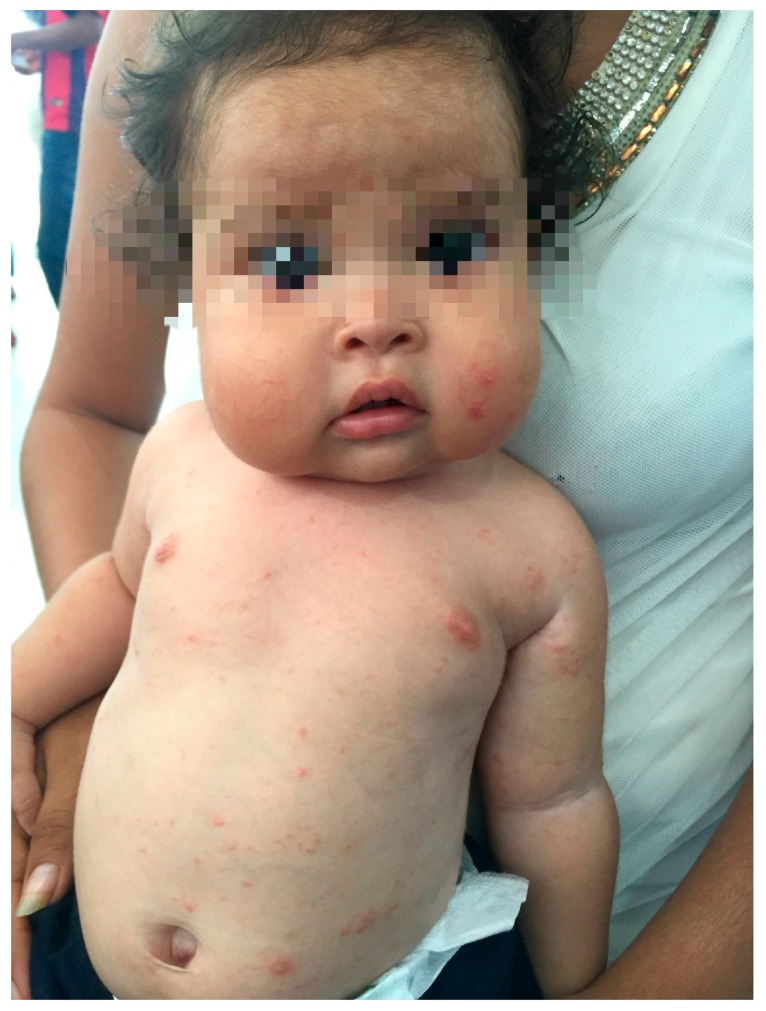
Prominent cheeks, mild telagiectasias and disseminated papulo-nodular lesions.

## Appendix A

**Table A1:** Cases of scabies and iatrogenic Cushing syndrome.

	Age (Months)	Sex	Topical Steroid Treatment			Clinical Features		
			Usage Duration	Frequency	Regularity	Adiposity	Scabies	Symptom Duration
1	5	Female	1 month	4 times a day	Continuous	+++	Crusted	1 month
2	6	Female	5 months	1–2 times a day	Intermittent	+++	Common	6 months
3	7	Male	3 months	2 times a day	Continuous	++++	Common	3 months
4	7	Male	2 months	2–3 times a day	Intermittent	+++	Common	7 months
5	8	Male	8 months	Daily	Continuous	++++	Crusted	8 months
6	9	Female	1 month	3 times a day	Continuous	++++	Common	6 months
7	16	Male	5 months	Daily	Continuous	++	Common	6 months
8	17	Female	4 months	1–2 times a day	Discontinuous	++	Common	3 months
